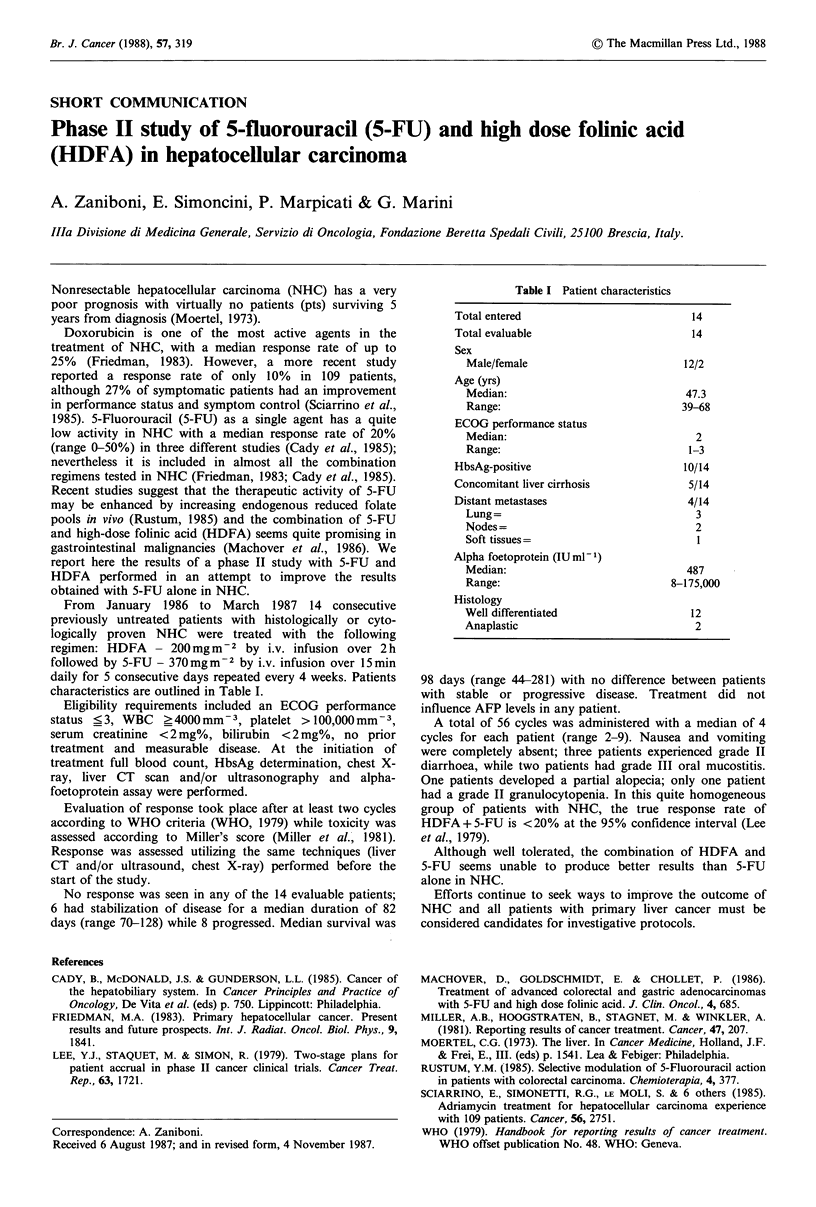# Phase II study of 5-fluorouracil (5-FU) and high dose folinic acid (HDFA) in hepatocellular carcinoma.

**DOI:** 10.1038/bjc.1988.71

**Published:** 1988-03

**Authors:** A. Zaniboni, E. Simoncini, P. Marpicati, G. Marini

**Affiliations:** IIIa Divisione di Medicina Generale, Fondazione Beretta Spedali Civili, Brescia, Italy.


 © The Macmillan Press Ltd., 1988

SHORT COMMUNICATION

### Phase II study of 5-fluorouracil (5-FU) and high dose folinic acid (HDFA) in hepatocellular carcinoma

A. Zaniboni, E. Simoncini, P. Marpicati & G. Marini

*IIIa Divisione di Medicina Generale, Servizio di Oncologia, Fondazione Beretta Spedali Civili, 25100 Brescia, Italy.*

Nonresectable hepatocellular carcinoma (NHC) has a very poor prognosis with virtually no patients (pts) surviving 5 years from diagnosis (Moertel, 1973).

Doxorubicin is one of the most active agents in the treatment of NHC, with a median response rate of up to 25% (Friedman, 1983). However, a more recent study reported a response rate of only 10% in 109 patients, although 27% of symptomatic patients had an improvement in performance status and symptom control (Sciarrino *et al.*, 1985). 5-Fluorouracil (5-FU) as a single agent has a quite low activity in NHC with a median response rate of 20% (range 0–50%) in three different studies (Cady *et al.*, 1985); nevertheless it is included in almost all the combination regimens tested in NHC (Friedman, 1983; Cady *et al.*, 1985). Recent studies suggest that the therapeutic activity of 5-FU may be enhanced by increasing endogenous reduced folate pools *in vivo* (Rustum, 1985) and the combination of 5-FU and high-dose folinic acid (HDFA) seems quite promising in gastrointestinal malignancies (Machover *et al.*, 1986). We report here the results of a phase II study with 5-FU and HDFA performed in an attempt to improve the results obtained with 5-FU alone in NHC.

From January 1986 to March 1987 14 consecutive previously untreated patients with histologically or cytologically proven NHC were treated with the following regimen: HDFA – 200 $mg m^{-2}$ by i.v. infusion over 2 h followed by 5-FU – 370 $mg m^{-2}$ by i.v. infusion over 15 min daily for 5 consecutive days repeated every 4 weeks. Patients characteristics are outlined in Table I.

Eligibility requirements included an ECOG performance status $\leqq 3$, WBC $\geqq 4000 mm^{-3}$, platelet $> 100,000 mm^{-3}$, serum creatinine $< 2$ mg%, bilirubin $< 2$ mg%, no prior treatment and measurable disease. At the initiation of treatment full blood count, HbsAg determination, chest X-ray, liver CT scan and/or ultrasonography and alpha-foetoprotein assay were performed.

Evaluation of response took place after at least two cycles according to WHO criteria (WHO, 1979) while toxicity was assessed according to Miller's score (Miller *et al.*, 1981). Response was assessed utilizing the same techniques (liver CT and/or ultrasound, chest X-ray) performed before the start of the study.

No response was seen in any of the 14 evaluable patients; 6 had stabilization of disease for a median duration of 82 days (range 70–128) while 8 progressed. Median survival was 98 days (range 44–281) with no difference between patients with stable or progressive disease. Treatment did not influence AFP levels in any patient.

**Table I** Patient characteristics

| | |
|---|---|
| Total entered | 14 |
| Total evaluable | 14 |
| Sex | |
| Male/female | 12/2 |
| Age (yrs) | |
| Median: | 47.3 |
| Range: | 39–68 |
| ECOG performance status | |
| Median: | 2 |
| Range: | 1–3 |
| HbsAg-positive | 10/14 |
| Concomitant liver cirrhosis | 5/14 |
| Distant metastases | 4/14 |
| Lung = | 3 |
| Nodes = | 2 |
| Soft tissues = | 1 |
| Alpha foetoprotein ($IU ml^{-1}$) | |
| Median: | 487 |
| Range: | 8–175,000 |
| Histology | |
| Well differentiated | 12 |
| Anaplastic | 2 |

A total of 56 cycles was administered with a median of 4 cycles for each patient (range 2–9). Nausea and vomiting were completely absent; three patients experienced grade II diarrhoea, while two patients had grade III oral mucostitis. One patients developed a partial alopecia; only one patient had a grade II granulocytopenia. In this quite homogeneous group of patients with NHC, the true response rate of HDFA + 5-FU is $< 20\%$ at the 95% confidence interval (Lee *et al.*, 1979).

Although well tolerated, the combination of HDFA and 5-FU seems unable to produce better results than 5-FU alone in NHC.

Efforts continue to seek ways to improve the outcome of NHC and all patients with primary liver cancer must be considered candidates for investigative protocols.

**References**

CADY, B., McDONALD, J.S. & GUNDERSON, L.L. (1985). Cancer of the hepatobiliary system. In *Cancer Principles and Practice of Oncology*, De Vita *et al.* (eds) p. 750. Lippincott: Philadelphia.

FRIEDMAN, M.A. (1983). Primary hepatocellular cancer. Present results and future prospects. *Int. J. Radiat. Oncol. Biol. Phys.*, **9**, 1841.

LEE, Y.J., STAQUET, M. & SIMON, R. (1979). Two-stage plans for patient accrual in phase II cancer clinical trials. *Cancer Treat. Rep.*, **63**, 1721.

MACHOVER, D., GOLDSCHMIDT, E. & CHOLLET, P. (1986). Treatment of advanced colorectal and gastric adenocarcinomas with 5-FU and high dose folinic acid. *J. Clin. Oncol.*, **4**, 685.

MILLER, A.B., HOOGSTRATEN, B., STAGNET, M. & WINKLER, A. (1981). Reporting results of cancer treatment. *Cancer*, **47**, 207.

MOERTEL, C.G. (1973). The liver. In *Cancer Medicine*, Holland, J.F. & Frei, E., III. (eds) p. 1541. Lea & Febiger: Philadelphia.

RUSTUM, Y.M. (1985). Selective modulation of 5-Fluorouracil action in patients with colorectal carcinoma. *Chemioterapia*, **4**, 377.

SCIARRINO, E., SIMONETTI, R.G., LE MOLI, S. & 6 others (1985). Adriamycin treatment for hepatocellular carcinoma experience with 109 patients. *Cancer*, **56**, 2751.

WHO (1979). *Handbook for reporting results of cancer treatment.* WHO offset publication No. 48. WHO: Geneva.

Correspondence: A. Zaniboni.
Received 6 August 1987; and in revised form, 4 November 1987.